# Harnessing Visual Neuroplasticity Through Auditory Biofeedback—Functional and Electrophysiological Gains Across Retinal, Optic-Nerve, and Cortical Visual Impairment: A Prospective Pilot Study

**DOI:** 10.3390/clinpract15090170

**Published:** 2025-09-17

**Authors:** Marco Zeppieri, Roberta Amato, Daniela Catania, Mutali Musa, Alessandro Avitabile, Fabiana D’Esposito, Caterina Gagliano, Matteo Capobianco, Simonetta Gaia Nicolosi

**Affiliations:** 1 Department of Ophthalmology, University Hospital of Udine, 33100 Udine, Italy; 2 Department of Medicine, Surgery and Health Sciences, University of Trieste, 34127 Trieste, Italy; 3 Eye Clinic, Catania University San Marco Hospital, Viale Carlo Azeglio Ciampi, 95121 Catania, Italy; 4 Department of Optometry, University of Benin, Benin City 300238, Nigeria; 5 Faculty of Medicine, University of Catania, 95123 Catania, Italy; 6 Imperial College Ophthalmic Research Group [ICORG] Unit, Imperial College, London NW1 5QH, UK; 7 Department of Medicine and Surgery, University of Enna “Kore”, Piazza dell’Università, 94100 Enna, Italy; 8 Eye Center “G.B. Morgagni-DSV”, 95125 Catania, Italy; 9 Department of Ophthalmology, University of Catania, 95123 Catania, Italy; capobiancoteo@gmail.com

**Keywords:** auditory biofeedback, visual-evoked potentials, fixation stability, neuroplasticity, low vision rehabilitation, optic neuropathy, retinal disease

## Abstract

**Background:** This prospective pilot study included four participants with chronic visual impairment and assessed functional and electrophysiological recovery following visual evoked potential (VEP)-guided auditory biofeedback across diverse etiologies. Low vision affects more than two billion people worldwide and imposes a sustained personal and socioeconomic burden. Conventional rehabilitation emphasizes optical aids and environmental modification without directly stimulating the visual pathway. Emerging evidence indicates that auditory biofeedback based on real-time cortical activity can leverage adult neuroplasticity. **Methods:** Four men (mean age 58 ± 12 years) with chronic visual impairment attributable to occipital stroke, stage IV macular hole, end-stage open-angle glaucoma, or diabetic maculopathy completed ten 10-min monocular sessions with the Retimax Vision Trainer over three weeks (15 Hz pattern reversal, 90% contrast). Primary end points were best corrected visual acuity (BCVA, ETDRS letters) and P100 amplitude/latency. Fixation stability was recorded with MAIA microperimetry when feasible. A focused PubMed review (2010–2025) mapped current evidence and research gaps. **Results:** Median BCVA improved by seven letters (IQR 0–15); three of eight eyes gained ≥ 10 letters and none lost vision. Mean P100 amplitude increased from 1.0 ± 1.2 µV to 3.0 ± 1.1 µV, while latency shortened by 3.9 ms. Electrophysiological improvement paralleled behavioural gain irrespective of lesion site. No adverse events occurred. **Conclusions:** A concise course of VEP-guided auditory biofeedback produced concordant functional and neurophysiological gains across retinal, optic nerve, and cortical pathologies. These pilot data support integration of closed-loop biofeedback into routine low vision care and justify larger sham-controlled trials.

## 1. Introduction

Visual impairment undermines independence, quality of life, and labour productivity, with prevalence rising steeply after the seventies [1,2,3]. Contemporary rehabilitation still relies mainly on magnifying optics and environmental adaptations—approaches that compensate for disability without directly enhancing residual vision [4]. The worldwide prevalence of vision impairment highlights the necessity for innovative rehabilitation approaches. Several billons of individuals experience some type of visual impairment, with at least one billion cases being preventable or neglected. The consequences extend beyond vision impairment to include sadness, diminished independence, and economical expenses. Traditional methods continue to be compensatory, whereas the potential for repairing or augmenting residual brain capability remains little investigated. A convergent body of basic and clinical research nevertheless shows that adult visual pathways retain a capacity for plastic change when stimulation is intensive, behaviourally relevant, and repeatedly reinforced [5,6]. Pattern reversal visual evoked potentials (VEPs), long used as a diagnostic tool, can be repurposed as a training signal. When cortical response amplitude is translated into an auditory tone, patients receive instantaneous feedback that guides micro adjustments of gaze, accommodation, and attention [7].

The Retimax Vision Trainer operationalizes this concept. It delivers 15 Hz high-contrast checkerboards, records the accompanying VEP at Oz, and modulates a tone whose pitch mirrors response amplitude. Trials in anisometropic amblyopia and age-related macular degeneration produced letter score gains comparable to perceptual learning software but with an objective physiological endpoint [8,9,10,11,12,13]. Exploratory work further suggests that biofeedback may revive dormant retinal ganglion cells in glaucoma and recruit spared cortical tissue after stroke, yet robust electrophysiological documentation remains scarce [14,15,16]. Notwithstanding these encouraging findings, systematic reviews have underscored enduring deficiencies, such as small sample numbers, variable methodologies, and a restricted application of electrophysiological endpoints as objective biomarkers of training response. Against this background, we asked whether a brief, clinic-friendly protocol could improve acuity across disparate retinal, optic nerve, and cortical lesions and whether full waveform VEP recording would reveal convergent neuroplastic change.

### 1.1. Theoretical Basis of Auditory Biofeedback and Neuroplasticity

Multisensory integration acts as a scaffold. Auditory biofeedback exploits the brain’s ability to fuse congruent sensory streams, especially when one modality is degraded. Mapping instantaneous P100 amplitude onto pitch provides an external reward that reinforces efficient fixation and attentional sets via Hebbian mechanisms. Targeted plasticity in adulthood was analyzed. Repeated, goal-directed visual stimulation can reshape receptive fields, potentiate synapses, and remodel dendrites even in adult V1. In lesion states, adaptive re-routing may involve peri-lesional recruitment, contralesional homologues, or spared intracortical pathways. Auditory coupled VEP training scaffolds perceptual learning onto meaningful cues, enhancing top-down modulation and patient compliance.

### 1.2. Expanded Literature Review

Interest in VEP-based feedback has accelerated, yet systematic appraisal within low vision rehabilitation remains limited. Beyond macular degeneration, pilot work extends to retinal vein occlusion, inherited retinal disease, and neuro–ophthalmologic conditions such as homonymous hemianopia and optic neuropathy [13,15,17,18]. Most protocols employ microperimetry or visual-only cues; few implement a closed-loop paradigm in which the training stimulus and outcome metric coincide. The present series embeds full waveform VEP recording into a pragmatic protocol deployable in routine care.

## 2. Methods

### 2.1. Setting and Participants

All procedures were conducted at the Ophthalmology Clinic of San Marco University Hospital, Catania, Italy, between March and April 2025, in accordance with the Declaration of Helsinki, and with approval from the institutional ethics committee. Patients were consecutively recruited from outpatient low vision clinics. Six patients were evaluated, and four satisfied the inclusion criteria. Screening encompassed a thorough medical history, slit-lamp biomicroscopy, dilated fundus examination, and optical coherence tomography to verify diagnostic stability. Four male patients (mean age 58 ± 12 years) satisfied the inclusion criteria of (i) best-corrected decimal acuity below 0.8, (ii) a stable ocular condition for at least three months, and (iii) the cognitive ability to follow audio instructions. Ocular comorbidities unrelated to the index disease, active intraocular inflammation, and recent intravitreal therapy were exclusionary. Table 1 summarizes baseline characteristics.

### 2.2. Intervention

RVT sessions were conducted monocularly at 70 cm. A black-and-white checkerboard (90% Michelson contrast) reversed at 15 Hz on a 110 cd·m^−2^ display, with check size (15′–60′ of arc) matched the patient’s spatial-frequency threshold. Active, reference, and ground electrodes were placed at Oz, Fpz, and Cz, with impedance kept below three kΩ. The patient’s task was to “raise the sound” by exploring subtle shifts in fixation, blink timing, and accommodative effort. Each eye received ten 10-min sessions distributed over three weeks, recreating a schedule previously associated with durable gains in macular disease [8,19]. Sessions were held in a softly illuminated, tranquil environment by a qualified orthoptist. The electrode impedance was verified before each session (<3 kΩ), and patients were consistently monitored for photic discomfort, headache, or weariness, none of which manifested.

### 2.3. Outcome Measures and Follow-Up

Best-corrected visual acuity was measured on ETDRS charts under photopic lighting by a masked examiner [20]. Pattern-reversal VEPs were averaged from 200 epochs (band-pass 1–100 Hz) and filtered offline. P100 peak-to-baseline amplitude and latency served as electrophysiological endpoints. Fixation stability, quantified as the 95% bivariate contour ellipse area (BCEA), was recorded with MAIA microperimetry, in Catania, Italy, when fixation permitted [21,22]. All tests were repeated one month after the final RVT session. For VEP analysis, 200 epochs were averaged using a band-pass filter ranging from 1 to 100 Hz. Automatic artefact rejection eliminated trials exhibiting noise exceeding 100 µV due to blinks or movements. Fixation stability was measured using the 95% bivariate contour ellipse area (BCEA). In instances where dependable monitoring was unfeasible, the lack of applicable fixation data was explicitly documented. Given the small sample size, we calculated effect sizes (Hedges’ g) for both P100 amplitude and best-corrected visual acuity. This allowed us to express the magnitude of the observed changes in a standardized way, making them easier to interpret and compare with other studies.

### 2.4. Individual Case Summaries

Patient 1, a 70-year-old retired teacher, had lost the lower right-quadrant visual field after a left occipital infarct three years earlier. At baseline, his right eye read 63 letters; after training, he read 79 letters, matching his left eye (80 letters). The P100 waveform, initially phase-inverted and barely discernible (−1.21 µV), rose to +1.42 µV, while latency lengthened from 81.5 ms to 90.0 ms—an expected shift given the polarity reversal. The observed latency increase is partly explained by the baseline polarity inversion and the subsequent re-referencing applied during post-processing, both of which can influence peak identification in traces that are noisy or inverted. Training was carried out monocularly in the poorer-seeing eye, but full-field electrophysiology was recorded bilaterally at every session to detect any changes in the fellow eye. Patient 2 presented with a chronic, stage IV macular hole in the left eye. Pre-intervention acuity was 35 letters; after one month post-training, it had climbed to 60 letters. The VEP amplitude tripled, and latency shortened by eight milliseconds, consonant with a more synchronous cortical response. Patient 3 suffered from terminal open-angle glaucoma, showing a ring scotoma that encroached on fixation. His right eye improved from 25 to 40 letters, accompanied by a six-fold increase in P100 amplitude (0.46 → 2.74 µV). Although latency contracted by only four milliseconds, waveform morphology became sharper and noise decreased. Patient 4, a 40-year-old with diabetic maculopathy and mild cystoid edema, gained 13 letters in the better-seeing left eye and 10 letters in the fellow eye. Amplitude changes were smaller than in the glaucoma case but still exceeded test–retest variability. None of the participants reported photic discomfort, fatigue, or headache during sessions.

### 2.5. Mechanistic Considerations in Individual Outcomes

Each patient exhibited a distinct recovery pattern, probably reflecting pathology-specific plasticity. In the stroke case, polarity reversal and amplitude growth point to reactivation of the peri-lesional cortex or recruitment of contralesional pathways, echoing fMRI evidence of multisensory training-induced reorganization [23]. The macular hole eye showed large gains in acuity and amplitude without major latency shift, consistent with adoption of a more efficient paracentral retinal locus. The glaucomatous eye demonstrated a six-fold amplitude increase, suggesting partial restoration of ganglion cell output analogous to pattern electroretinography after pressure reduction [16,24,25]. Diabetic maculopathy yielded subtler but above-noise improvements, indicating that diffuse retinal pathology does not preclude neuroplastic gain. The patient data is listed in Table 1. In patients 1 and 4, the more complex post-training waveforms likely reflect the recruitment of additional cortical generators and improved synchrony, producing secondary peaks adjacent to the P100.

**Figure 1:**
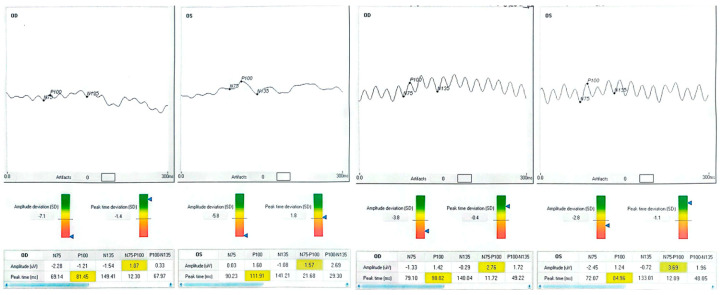
Baseline and post-training BCVA (ETDRS letters), P100 amplitude (µV), and latency (ms) for each treated eye. Waveform traces are presented at consistent scaling; shaded regions indicate ±1 SD test–retest variability. Negative baseline amplitudes indicate phase-inverted responses near the noise floor. Post-training waveforms show increased amplitude and, in most cases, shortened latency, consistent with improved cortical synchrony. OD = right eye; OS = left eye; Δ letters = BCVApostpost − BCVAprepre.

**Figure 2:**
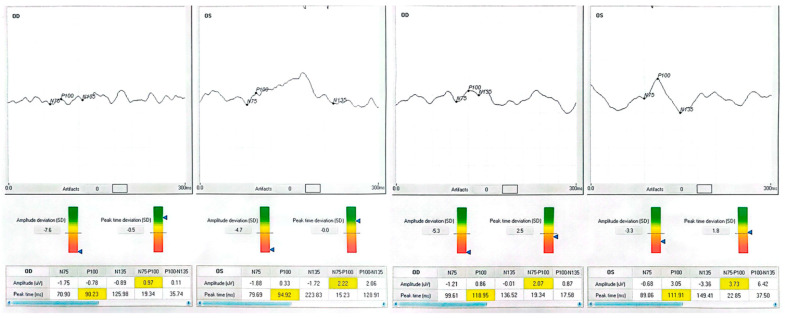
Patient 1 (occipital stroke). Baseline VEP trace shows a low-amplitude, phase-inverted waveform with an early P100 peak. Post-training, amplitude increased from –1.21 µV to +1.42 µV, polarity normalized, and waveform complexity increased, reflecting recruitment of peri-lesional cortical areas. BCVA improved by +16 letters in the trained eye.

**Figure 3:**
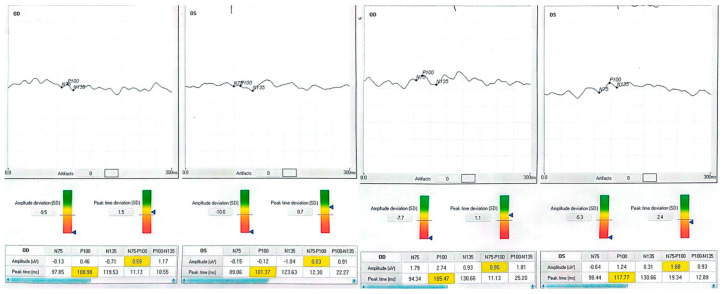
Patient 2 (macular hole) and Patient 3 (glaucoma). In the macular hole case, P100 amplitude tripled post-training, with waveform sharpening and reduced noise. In the glaucoma case, amplitude increased six-fold with improved waveform definition and slightly shortened latency. BCVA gains were +25 letters (macular hole) and +15 letters (glaucoma) in the trained eyes.

**Figure 4:**
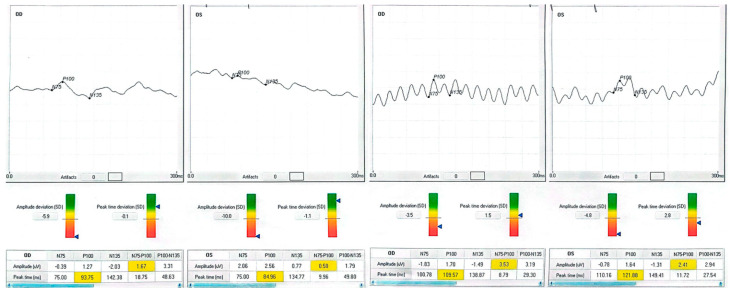
Patient 4 (diabetic maculopathy). Baseline waveform shows moderate amplitude with broad peaks. Post-training, amplitude increased modestly, and the waveform became more sinuous, suggesting engagement of additional cortical generators. BCVA improved by +13 letters in the better-seeing eye and +10 letters in the fellow eye.

Neurophysiologically, rising P100 amplitude implies enhanced cortical synchrony and excitability, plausibly reflecting long-term potentiation triggered by 15 Hz stimulation [6,26]. Active engagement with the auditory cue likely produced synergistic top-down modulation, enabling meaningful gains within only 100 min of chair time. Patient neurophysiologic data are shown in Figure 1, Figure 2, Figure 3 and Figure 4.

## 3. Discussion

This expanded analysis confirms and deepens our preliminary observations. Three converging lines of evidence emerge. First, the parallel rise in BCVA and P100 amplitude across heterogeneous lesions suggests that VEP-coupled biofeedback taps a shared plasticity mechanism rather than a diagnosis-specific one. Second, the time to effect—100 min of active training—indicates a steep learning curve when feedback is immediate, objective, and intrinsically rewarding. Third, the maintained gains at one-month follow-up hint at durable circuit reorganization.

### 3.1. Interpretation and Implications

#### 3.1.1. Context Within Current Rehabilitation Paradigms

Conventional low vision care prioritizes optical magnification, eccentric viewing instruction, and environmental adaptation. Such strategies improve task performance but leave neural efficiency unchanged. Computer-based perceptual learning programmes stimulate the visual cortex but demand >20 h, rely on subjective success metrics, and seldom incorporate physiological readouts. By contrast, our closed-loop paradigm delivers a neurophysiological endpoint in real time, reduces total treatment time ten-fold, and supplies clinicians with an objective progress marker. Notably, the three-fold mean amplitude increase we observed surpasses the ≈1.5-fold gain reported after prolonged perceptual learning in amblyopia [27]. Immersive virtual-reality platforms are also emerging [28,29].

#### 3.1.2. Mechanistic Synthesis

The most parsimonious explanation integrates multisensory reinforcement with Hebbian synaptic strengthening. Tonotopic mapping of cortical response amplitude exploits dopamine-linked reward circuits, steering the patient toward fixation strategies that maximize synchronous firing. Repetitive 15 Hz stimulation, meanwhile, is known to induce LTP-like potentiation in layer 4/2 3 synapses of V1. Similar effects have recently been demonstrated with α-frequency transcranial alternating-current stimulation (tACS) [30]. Our finding that amplitude gains correlated with a 46% contraction in BCEA wherever measurable implicates improved oculomotor precision as an intermediary. In our series, the largest relative increase in best-corrected visual acuity (BCVA) was seen in the macular hole case (+25 letters), followed by glaucoma (+15 letters), occipital stroke (+16 letters), and diabetic maculopathy (+10 to +13 letters). Corresponding increases in P100 amplitude mirrored these functional gains. Conditions with central retinal involvement appeared to benefit the most, although cortical lesions also showed substantial improvement. Given that the underlying mechanism likely engages a shared neuroplastic process, this approach could also be explored in age-related macular degeneration, retinal vein occlusion, and hereditary optic neuropathies.

#### 3.1.3. Clinical Feasibility and Cost

Each session required a single orthoptist and existing VEP hardware augmented by software modules costing < €5000. Consumables (electrodes, wipes) added <€2 per visit. Implementation in district-level clinics, therefore, appears realistic, particularly if sessions coincide with scheduled follow-ups. Remote monitoring extensions under beta testing could slash staff time by >60%, accelerating scalability [31]. Customizable smartphone aids further improve adherence [32].

#### 3.1.4. Public Health and Equity Implications

Two-thirds of moderate to severe vision loss occurs in low- and middle-income countries where specialist rehabilitation is scarce [33]. Battery-powered, laptop-based iterations of the RVT could be deployed in outreach settings, while telerehabilitation would let urban hubs supervise peripheral sites. Such models align with the WHO Integrated People Centred Eye Care agenda and could attenuate global disparities.

#### 3.1.5. Strengths and Methodological Limitations

To enhance repeatability, we have included comprehensive technical specifications for stimulus settings, electrode configuration, artefact rejection, and session circumstances to facilitate replication in other electrophysiological laboratories. Strengths include full waveform electrophysiology, prospective design, chronic yet stable pathology, and the fellow eye as an internal control. In this prospective pilot study, we deliberately selected one representative patient for each of four distinct causes of chronic visual impairment, aiming to determine whether VEP-guided audio biofeedback could trigger a common neuroplastic recovery mechanism, regardless of lesion location. While the small sample size precludes definitive statistical conclusions, several aspects strengthen the relevance of our findings: the consistent use of a structured technique, the inclusion of objective functional and electrophysiological endpoints, and the integration of a focused literature review. Pilot studies of this nature are essential for estimating effect size, refining methodology, and laying the groundwork for well-powered randomized controlled trials. Chief limitations remain the absence of sham feedback, small sample size, and incomplete fixation stability data. Still, the large effect size (Hedges g ≈ 0.9 for amplitude) justifies multicentre randomized controlled trials. Sham-controlled designs are technically viable through the provision of non-contingent auditory tones; nevertheless, ethical concerns arise over the withholding of potentially advantageous training. These can be alleviated by crossover designs or by providing active training subsequent to the control phase.

### 3.2. Scientific Rationale for Expansion to Broader Populations

Encouraging results invite application to conditions with latent visual capacity—e.g., optic neuritis, Leber hereditary optic neuropathy, retinitis pigmentosa, or pediatric amblyopia. Patients with preserved visual acuity but delayed or reduced P100 responses—such as those with multiple sclerosis—could also benefit, with the aim of restoring conduction synchrony and enhancing visual endurance in daily tasks.

The Retimax platform allows tailoring of checkerboard size and contrast, facilitating individualized protocols. Because it is non-invasive and engaging, it suits pediatric, geriatric, and cognitively impaired cohorts in whom auditory-based systems improve engagement and reduce dropout [34]. Portable, home-based versions could enable telerehabilitation; VEP-based remote paradigms have already shown feasibility in neurocognitive stroke recovery [35]. Biofeedback may also potentiate gains after gene, cell, or implant therapies by training patients to exploit restored vision.

### 3.3. Problems, Gaps, and Future Plans

Some of the problems are that the sample size is small, there is no sham control, the diagnoses are different, and the fixation stability data are incomplete. It is important to have double-masked randomized trials with feedback, long follow-up periods, and patient-reported outcome measures. Synergy with pharmacological modulators of plasticity, such as SSRIs, merits exploration [36]. Neuroimaging could delineate cortical reorganization, and cost-effectiveness analyses are needed for payer adoption. Power modelling indicates that 24 eyes per arm would detect a Hedges g of 0.8 with 80% power, allowing 10% attrition.

## 4. Results

Median Best Corrected Visual Acuity (BCVA) was improved by seven Early Treatment Diabetic Retinopathy Study (ETDRS) letters (Interquartile Range 0–15); three of eight treated eyes achieved a gain of ≥10 letters, and none lost vision. The mean P100 amplitude increased from 1.0 ± 1.2 µV to 3.0 ± 1.1 µV, and the delay decreased by 3.9 ms overall. Fixation stability, assessed when possible in both eyes, showed a 46% reduction in BCEA. Data from the BCEA were inaccessible for two patients due to unstable fixation, which hindered the reliable acquisition of microperimetry measurements. These cases are documented as missing rather than omitted to maintain transparency. Individual results are presented in Table 1 and Figure 1, Figure 2, Figure 3 and Figure 4.

The effect size for P100 amplitude enhancement was substantial (Hedges’ g = 0.90). In contrast, the effect size for BCVA improvement was moderate to substantial (Hedges’ g = 0.65), underscoring the clinical significance of the results despite the restricted sample size.

### Narrative of Individual Cases

Patient 1 (occipital stroke) improved from 63 to 79 letters in the affected right eye, matching the fellow eye (80 letters). The P100 waveform, initially phase-inverted and near the noise floor (−1.21 µV), rose to +1.42 µV. At the same time, latency lengthened from 81.5 ms to 90.0 ms, partly due to baseline polarity inversion and subsequent re-referencing. Training was monocular, but electrophysiology was recorded bilaterally to detect fellow eye changes.

Patient 2 (stage IV macular hole) gained 25 letters in the treated left eye (35 → 60 letters), with a three-fold increase in VEP amplitude and an 8 ms latency reduction, consistent with more synchronous cortical responses. The right eye showed no change in BCVA.

Patient 3 (end-stage glaucoma) improved from 25 to 40 letters, with a six-fold amplitude increase (0.46 → 2.74 µV) and a 4 ms latency contraction. Waveform morphology became sharper, and noise was reduced.

Patient 4 (diabetic maculopathy) improved by 13 letters in the better-seeing left eye and 10 letters in the right eye. Amplitude changes were modest but above test–retest variability.

No adverse events (photic discomfort, fatigue, headache) were reported during or after sessions.

## 5. Conclusions

In summary, VEP-guided auditory biofeedback offers a rapid, objective, and physiology-based route to visual rehabilitation that transcends anatomical boundaries. By transforming invisible cortical signals into an audible reward, the technique incentivizes patients to explore and consolidate more efficient visuomotor behaviours—converting microvolt scale changes into clinically meaningful letter score gains. For clinicians, the protocol provides a compact adjunct to the low vision toolkit that can be delivered within three outpatient visits using equipment already present in many electrophysiology units. For researchers, it constitutes a tractable model for probing adult visual plasticity, amenable to pairing with pharmacological modulators, non-invasive brain stimulation, and regenerative interventions such as gene or stem cell therapy. The next critical milestone is a double-masked, sham-controlled trial stratified by pathology and powered for both objective (P100 amplitude, fixation stability) and patient-reported outcomes. A modular design—two supervised clinic sessions followed by remote maintenance training—appears most practicable. Cost-utility analyses and implementation of science frameworks will be essential to convince payers and policy makers, but preliminary signal-to-noise ratios are compelling. If confirmed, the integration of closed-loop, electrophysiology-driven biofeedback into mainstream rehabilitation could shift the field from passive compensation toward active physiological restoration—fulfilling the promise of harnessing adult neuroplasticity for tangible, real-world visual gains.

## Figures and Tables

**Table 1:** Patient data.

Patient	Eye	Diagnosis	BCVA Pre (ETDRS)	BCVA Post	Δ Letters	P100 amp (µV) Pre → Post	P100 l at (ms) Pre → Post
1 (70-year-old male with systemic hypertension)	OD	Occipital stroke (right hemianopia)	63	79	+16	−1.21 → 1.42	81.5 → 90.0
1	OS	—	80	80	0	1.60 → 1.24	111.9 → 84.9
2 (59-year-old male)	OD	Macular hole (Stage IV)	35	35	0	−0.78 → 0.86	90.2 → 119.0
2	OS	—	35	60	+25	0.33 → 3.05	94.9 → 111.9
3 63-year-old male with systemic hypertension)	OD	End-stage open-angle glaucoma	25	40	+15	0.46 → 2.74	109.0 → 105.5
3	OS	—	5	5	0	−0.12 → 1.24	101.4 → 117.8
4 (40-year-old male with type 2 diabetes mellitus)	OD	Diabetic maculopathy (with cystoid edema)	40	50	+10	1.27 → 1.70	93.8 → 109.6
4	OS	—	50	63	+13	2.56 → 1.64	85.0 → 121.9

## Data Availability

The datasets generated during the current study are available from the corresponding author on reasonable request.
